# Metagenomic Microbial Signatures for Noninvasive Detection of Pancreatic Cancer

**DOI:** 10.3390/biomedicines13041000

**Published:** 2025-04-21

**Authors:** Yueying Chen, Fulin Nian, Jia Chen, Qiuyu Jiang, Tianli Yuan, Haokang Feng, Xizhong Shen, Ling Dong

**Affiliations:** 1 Department of Gastroenterology and Hepatology, Zhongshan Hospital, Fudan University, Shanghai 200032, China; 2 Shanghai Institute of Liver Diseases, Shanghai 200032, China; 3 Department of Gastrointestinal Surgery, Renji Hospital Affiliated, University School of Medicine, Shanghai Jiao Tong University, Shanghai 200127, China; 4 Key Laboratory of Carcinogenesis and Cancer Invasion, Department of Liver Surgery, Liver Cancer Institute, Zhongshan Hospital, Fudan University, Ministry of Education, Shanghai 200032, China

**Keywords:** pancreatic ductal adenocarcinoma, diagnostic biomarker, microbial signatures, shotgun metagenomics, CA19-9

## Abstract

**Background/Objectives:** Pancreatic ductal adenocarcinoma (PDAC) is a highly aggressive malignancy with poor early detection rates owing to the limited sensitivity and specificity of the current biomarker CA19-9. Gut microbiota dysbiosis plays a key role in PDAC pathogenesis. This study aimed to evaluate the noninvasive approach we developed, combining metagenome-derived microbial signatures with CA19-9, to improve PDAC detection. **Methods:** This study included 50 treatment-naïve patients with PDAC and their matched controls. Fecal samples were analyzed using shotgun metagenomic sequencing. Machine learning algorithms were used to develop and validate a diagnostic panel integrating microbial signatures and CA19-9 levels. Subgroup analyses were used to confirm the robustness of the microbial markers. **Results:** The combined models at both species and genus levels effectively distinguished patients with PDAC from healthy individuals, and their strong diagnostic efficacy and accuracy were demonstrated through rigorous validation. **Conclusions:** In conclusion, the combination of gut microbiome profiling and CA19-9 improves PDAC detection accuracy compared to the use of CA19-9 alone, showing promise for early and noninvasive diagnosis.

## 1. Introduction

Global epidemiological data reveal a concerning upward trajectory in pancreatic ductal adenocarcinoma (PDAC) incidence, positioning this malignancy to become the second most lethal cancer worldwide by 2025 [1,2]. The highly aggressive nature of PDAC directly contributes to its dismal prognosis, with a 5-year survival rate below 10% [3,4]. Currently, surgery is the sole potentially curative approach for PDAC. However, only about 20% of patients are diagnosed at an early stage with resectable lesions [5]. Previous studies have identified several risk factors for PDAC, including smoking, alcohol consumption, obesity, periodontal disease, and diabetes [3]. Moreover, numerous potential biomarkers for pancreatic cancer have been discovered in blood and tumor tissues [6]. Despite these advancements, the early and accurate diagnosis of PDAC remains a significant challenge, underscoring the urgent need for more reliable diagnostic tools and strategies.

As the most commonly used diagnostic indicator for PDAC, the elevation of serum CA19-9 might be affected by lifestyle factors, such as excessive tea consumption [7], and is associated with various pathological conditions, including liver damage, biliary obstruction and inflammation, pancreatitis, diabetes [8]. Additionally, CA19-9 lacks tumor-type specificity, as its elevation can be observed in multiple malignancies, including those originating from colorectum, stomach, lung, breast, liver, and pancreatic neuroendocrine tumors [9,10]. Therefore, identifying more effective markers for an accurate and early diagnosis of PDAC is essential.

Emerging evidence positions the gut microbiome as a pivotal modulator of oncogenesis through mechanisms involving the induction of chronic inflammation, immune system modulation, and secretion of specific microbial metabolites [11]. Although inflammation is a protective response, it can also become a risk factor for the development of cancers. Microbiome-activated inflammatory responses such as pancreatic cancer cells exposed to *Enterococcus faecalis* showed an increased expression of pro-inflammatory cytokines C-X-C motif ligand 8 (CXCL8) and vascular endothelial-derived growth factor (VEGF), which promote fibrosis and angiogenesis [12]. The immune modulation by the microbiome has been confirmed. The ablation of microbiota accelerated the Th1 polarization of CD4^+^ T cells, enhanced the cytotoxic phenotype of CD8^+^ T cells, and increased the expression of T-BET, TNF-α, and IFN-γ [13]. In addition to microbial involvement in the development of cancer, microbial metabolites can directly shape host and tumor metabolism, induce cell damage, and regulate tumor immunity. Butyrate is a short-chain fatty acid (SCFA) produced by the bacterial metabolism of dietary fiber, which has been proven to have antitumor effects and was associated with decreased colorectal cancer incidence [14]. Butyrate has been shown to reduce the proliferation of PDAC cells and induce their differentiation to a secretory phenotype [15]. The bacterial metabolite deoxycholic acid could promote cell cycle progression by stimulating EGFR, MAPK, and STAT3 signaling in pancreatic cancer cells [16]. In contrast, ursodeoxycholic acid has been shown to have anticancer effects in pancreatic cancer cell lines by inhibiting epithelial–mesenchymal transformation [17]. Trimethylamine N-oxide (TMAO) is a product converted from dietary choline by gut bacterial enzymes, which could induce inflammation and immune activation. A recent study suggested that TMAO could drive antitumor immunity by inducing the immunostimulatory phenotype of macrophages and enhancing the function of effector T cells [18].

In PDAC, dysbiosis patterns correlate with disease progression, suggesting microbial signatures could serve as novel screening tools [19]. Seminal work by Gophna et al. demonstrated PDAC-associated enrichment of *Proteobacteria*, *Akkermansia*, and *Veillonella*, contrasted by depletion of *Lachnospiraceae* and Ruminococcaceae in healthy controls. Their machine learning model leveraging these taxa achieved an AUC of 0.825, validating microbiota-based diagnostic potential [20]. Such advancements align with broader applications of artificial intelligence in oncology for risk stratification and outcome prediction, supporting integrative approaches to refine PDAC detection [21,22].

In this study, we present a systematic investigation combining shotgun metagenomic profiling of fecal microbiota with machine learning to develop a noninvasive PDAC diagnostic framework. By analyzing samples from 50 treatment-naïve PDAC patients and 50 matched controls, we delineated disease-specific microbial signatures across taxonomic ranks and constructed robust classifiers outperforming CA19-9 alone. We established combinatorial models integrating metagenomic features with CA19-9 to maximize diagnostic precision. By characterizing gut microbial signatures specific to PDAC, this work addresses a fundamental diagnostic gap in current clinical practice and pioneers a novel microbiome-based detection paradigm.

## 2. Methods

### 2.1. Study Design and Sample Collection

This study adhered to the STARD guidelines and enrolled 50 patients with PDAC and 50 matched healthy controls from October 2023 to January 2024. Healthy controls were age- and sex-matched to patients with PDAC, with additional screening to ensure comparable habitual diets (assessed by a simplified food frequency questionnaire). Individuals reporting extreme dietary patterns (e.g., vegetarian, ketogenic, or high-fat diets) or the use of antibiotics, probiotics, or immunosuppressive agents within the past 3 months were excluded. Fecal samples were collected in sterile tubes with 2% glycerol and stored at −80 °C within one hour. This study was approved by the Institutional Review Board of Fudan University Affiliated Zhongshan Hospital (Approval No. B2023-282R), and written informed consent was obtained from all participants.

### 2.2. Shotgun Metagenomic Sequencing and Data Analysis

Shotgun metagenomic sequencing was performed using the Illumina NovaSeq™ X Plus platform in paired-end mode. DNA was fragmented to 400 bp, and libraries were prepared using the NEXTFLEX Rapid DNA-Seq kit. Raw data were deposited in the NCBI Short Read Archive. The raw sequencing reads were trimmed of adapters, and low-quality reads (length < 50 bp or with a quality value < 20 or having N bases) were removed by fastp (https://github.com/OpenGene/fastp, version 0.23.0, accessed on 1 October 2024). Reads were aligned to the homo sapiens genome by BWA (http://bio-bwa.sourceforge.net, version 0.7.17, accessed on 1 October 2024), and any hit associated with the reads and their mated reads were removed. High-quality reads were assembled with MEGAHIT with default parameters, and ORFs ≥ 100 bp were predicted using Prodigal. A non-redundant gene catalog was constructed with CD-HIT (90% identity, 90% coverage), and gene abundance was estimated using SOAPaligner. Taxonomic annotation was performed using DIAMOND against the NCBI NR database (e-value < 1 × 10^−5^). Differential analysis was conducted using the Wilcoxon Rank-Sum Test.

### 2.3. Microbial Signature Selection and Model Construction

To ensure a balanced representation across subgroups, we employed stratified random sampling to partition the dataset into training and validation sets at a ratio of 6:4. This partitioning was stratified by age, sex, and TNM stage using the createDataPartition function from the caret package in R. The RPKM (Reads Per Kilobase of transcript, per Million mapped reads) values of differentially abundant microbial taxa were log10-transformed to mitigate right-skewness in microbial count distributions and standardized using z-score transformation to normalize feature scales for machine learning algorithms. In the training set, least absolute shrinkage and selection operator (LASSO) regression was initially applied for variable selection to identify the most relevant microbial features. LASSO regression was performed using the glmnet package in R. The optimal lambda value was selected from 100 candidates logarithmically spaced between 10^−5^ and 10^0^, using 10-fold cross-validation to minimize the cross-validation error. This step identified a subset of microbial features with non-zero regression coefficients. Following this, random forest analysis was performed to evaluate the importance of all variables, including both the LASSO-selected features and other potential predictors. The random forest model was constructed using the caret package in R, with model parameters optimized through 10-fold repeated cross-validation with 5 repeats. The number of trees in the random forest was set to 500, and the feature subset size at each split was set to the square root of the total number of features, which is the default behavior for classification tasks in random forests. Microbial features with non-zero regression coefficients from LASSO and importance values exceeding 0.2 in the random forest analysis were selected as the final set of predictors for the model. The final predictive model was constructed using a random forest classifier implemented via the R caret package, trained on microbial features selected through LASSO regression (non-zero coefficients) and random forest importance scoring (>0.2 threshold). The random forest model was configured with 500 decision trees (ntree) and utilized Gini impurity minimization for node splitting, with the optimal number of features per split (mtry) determined through 5 repeats of 10-fold cross-validation. This approach aimed to optimize model generalizability and minimize overfitting, thereby enhancing the predictive accuracy of the microbial-signature-based classification model.

### 2.4. Validation of Predictive Model Performance

The discrimination performance of the microbial classifiers was evaluated using receiver operating characteristic (ROC) curve analysis, with the AUC serving as a key metric for assessing predictive accuracy. Calibration curves were generated to evaluate the model’s calibration by comparing predicted probabilities with observed outcomes, ensuring the reliability of the model’s probability estimates. Clinical utility was further examined using decision curve analysis (DCA) to quantify the net benefit of the model across different threshold probabilities. To compare predictive performance, the AUC and accuracy of the microbial signature combined with CA19-9 levels were assessed against those of CA19-9 alone across all samples, providing a comprehensive evaluation of the added value of microbial features. Additionally, stratified analyses were conducted based on age, sex, BMI, and tumor stage to validate the robustness and generalizability of the microbial model across diverse subgroups.

### 2.5. Statistical Analysis

Continuous variables were analyzed using Student’s *t*-test for normally distributed data or the Mann–Whitney U test for non-normally distributed data, with results expressed as mean ± standard deviation (SD) or median (interquartile range), respectively. Categorical variables were evaluated using the χ^2^ test, with outcomes reported as frequencies and percentages. ROC curves were generated to assess diagnostic performance, and the AUC values along with 95% confidence intervals (CIs) were computed using the pROC package in R. Bootstrap resampling with 2000 replicates was employed to estimate the CIs. The optimal cutoff points for the ROC curves were identified using Youden’s index. Comparisons between ROC curves were performed using the Delong test. The sensitivity, specificity, and accuracy of the microbial signatures were calculated across all cohorts using the pROC package. A significance threshold of *p* < 0.05 was applied for all statistical tests. Analyses were conducted using R (version 4.2.1) and GraphPad Prism 9 (La Jolla, CA, USA).

## 3. Results

### 3.1. Microbiota and Functional Features of PDAC

**Figure 1:**
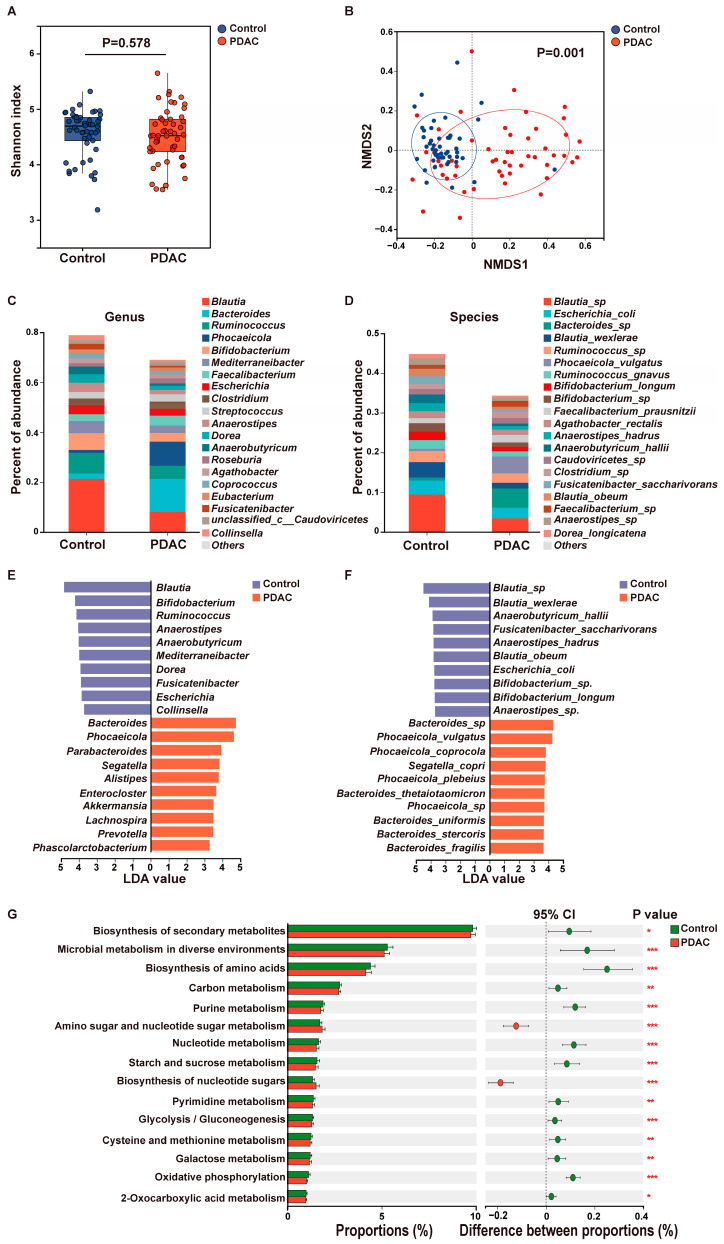
Comparison of the fecal microbiomes between controls and PDACs. (**A**) Shannon index of genus abundance in controls and patients with PDAC. Numbers in the plots show *p* values obtained from the Wilcoxon rank−sum test. n = 50 per group. (**B**) NMDS plots showing the similarity of the samples. Red and blue circles represent PDAC and controls, respectively. *p* values were obtained from permutational analysis of variance. (**C**,**D**) The composition of gut microbiota between healthy people and PDACs at the genus (**C**) and species level (**D**). (**E**,**F**) LEfSe analysis of significantly differential microbiota between healthy people and PDACs at the genus (**E**) and species level (**F**). (**G**) The bar chart displays the differences in metabolic pathways of microbial communities between the control group and the PDAC group. *p* values were obtained from the Wilcoxon rank-sum test. * *p* < 0.05, ** *p* < 0.01, *** *p* < 0.001.

Fecal samples were collected from 50 treatment-naïve PDAC patients and 50 healthy controls, with the two groups demonstrating balanced baseline characteristics (Table 1). The PDAC cohort included patients with early-stage (I [n = 19] and II [n = 11]) and advanced-stage (III [n = 12] and IV [n = 8]) disease. Shotgun metagenomic analysis identified 17,748 and 15,011 microbial species in the PDAC and control groups, respectively. Although no significant difference in α-diversity, as measured by the Shannon index (*p* = 0.578), was observed between patients and controls, suggesting comparable overall microbial richness and evenness in the gut microbiota of PDAC patients and healthy individuals (Figure 1A), β-diversity analysis revealed a significant distinction in microbial community structures (*p* = 0.001). This indicates substantial variation in the composition and relative abundance of microbial species between the two groups (Figure 1B). These findings suggest that specific microbial taxa or functional profiles, rather than overall diversity, may be critically implicated in the development or progression of PDAC.

A comparison of microbial composition between the two groups revealed significant differences at both the genus and species levels. At the genus level, healthy controls exhibited higher abundances of *Blautia, Ruminococcus,* and *Bifidobacterium*, whereas patients with PDAC showed increased abundances of *Bacteroides* and *Phocaeicola* (Figure 1C). At the species level, controls were enriched in *Blautia wexlerae*, *Blautia_obeum*, *Bifidobacterium_longum*, and *Escherichia coli*, while *Bacteroides sp.* and *Phocaeicola vulgatus* were more abundant in the PDAC group (Figure 1D). LEfSe analysis further identified microbial signatures with significant abundance differences between the two groups. Healthy controls were enriched in beneficial species such as *Blautia wexlerae*, *Blautia obeum*, and *Bifidobacterium longum*, which may play protective roles against tumorigenesis [23,24,25]. In contrast, patients with PDAC showed significant enrichment of *Bacteroides* and *Phocaeicola* species, including *Bacteroides thetaiotaomicron*, *Bacteroides uniformis*, *Bacteroides stercoris*, *Bacteroides fragilis*, *Phocaeicola vulgatus*, *Phocaeicola coprocola*, and *Phocaeicola plebeius*. In PDAC tumor tissues, a higher abundance of *Bacteroides* is associated with poor prognosis in patients, and compared with benign diseases, patients with PDAC exhibit an increased abundance of *Bacteroides* in bile samples [26,27]. Studies have reported that *Bacteroides* may promote the progression of colorectal cancer through mechanisms such as activating the NF-kB signaling pathway, accumulating regulatory T cells to enhance inflammatory responses, causing DNA damage via polyamine metabolism, and disturbing the host immune apparatus and gut barrier [28]. Similarly, *Phocaeicola* is also associated with a higher incidence of colorectal cancer [29]. However, its roles in PDAC progression remain complex and dualistic (Figure 1E,F).

Functional analysis revealed distinct metabolic pathway profiles between the groups. Pathways such as amino sugar and nucleotide sugar metabolism and biosynthesis of nucleotide sugars were significantly enriched in patients with PDAC, while biosynthesis of secondary metabolites, microbial metabolism in diverse environments, biosynthesis of amino acids, purine metabolism, and nucleotide metabolism were more prevalent in controls (Figure 1G). These findings demonstrated distinct fecal microbial profiles and functions between patients with PDAC and controls, suggesting that specific microbial taxa and metabolic pathways may be associated with PDAC progression.

### 3.2. Construction and Validation of Metagenomic-Based PDAC Classifiers

**Figure 2:**
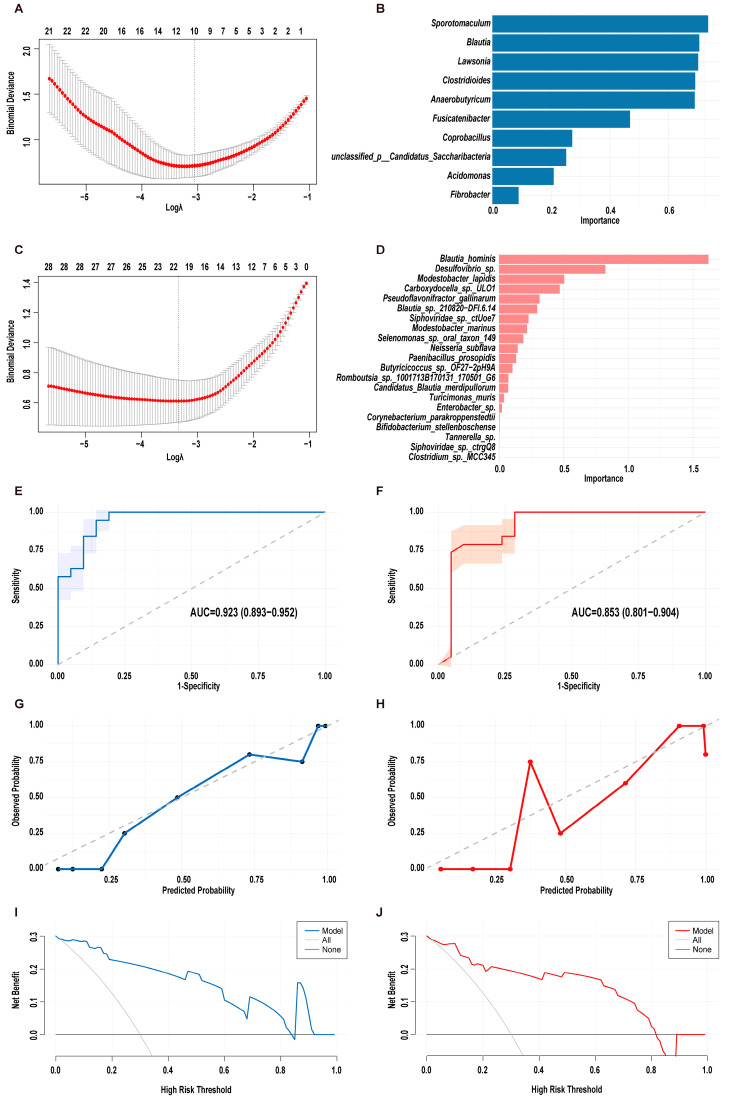
Construction and validation of metagenomic-based PDAC classifiers. (**A**) Microbial signature selection at the genus level in the LASSO model using ten-fold cross-validation. Dotted vertical lines were drawn at the optimal values by using the minimum deviance criteria. (**B**) The bar chart displays the importance of the 10 genera with non-zero coefficients in the LASSO analysis. (**C**) Microbial signature selection at the species level in the LASSO model using ten-fold cross-validation. Dotted vertical lines were drawn at the optimal values by using the minimum deviance criteria. (**D**) The bar chart displays the importance of the 21 bacterial species with non-zero coefficients in the LASSO analysis. (**E**,**F**) The ROC curves of model 1 (**E**) and model 2 (**F**) constructed at the genus and species levels. (**G**,**H**) Calibration plots of model 1 (**G**) and model 2 (**H**) constructed at the genus and species levels. (**I**,**J**) DCA of plots of model 1 (**I**) and model 2 (**J**) constructed at the genus and species levels. LASSO, least absolute shrinkage and selection operator; ROC, receiver operating characteristic; AUC, area under the curve; DCA, decision curve analysis.

The Wilcoxon rank-sum test demonstrated significant disparities in microbial composition between the control group and patients with PDAC, revealing 98 differentially abundant genera and 1935 differentially abundant species. In the training set, LASSO regression initially identified 10 potential microbial features at the genus level (Figure 2A). Subsequent analysis using random forest pinpointed nine genera with importance values exceeding 0.2, namely *Sporotomaculum*, *Blautia*, *Lawsonia*, *Clostridioides*, *Anaerobutyricum, Fusicatenibacter*, *Coprobacillus*, *unclassified_p__Candidatus_Saccharibacteria*, and *Acidomonas* (Figure 2B). At the species level, LASSO regression highlighted 21 species with non-zero coefficients (Figure 2C), from which 8 species were ultimately selected for final model construction: *Blautia_hominis*, *Desulfovibrio_sp.*, *Modestobacter_lapidis*, *Carboxydocella_sp._ULO1*, *Pseudoflavonifractor_gallinarum*, *Blautia_sp._210820-DFI.6.14*, *Siphoviridae_sp._ctUoe7*, and *Modestobacter_marinus* (Figure 2D). These microbial features hold promise as potential diagnostic biomarkers for PDAC. Consequently, two distinct random forest models were established: model 1 comprising the nine genera and model 2 incorporating the eight species.

Both microbial signature models demonstrated robust diagnostic capabilities in the validation cohort, with model 1 exhibiting superior discriminative power (AUC = 0.923, 95% CI: 0.893–0.952) compared to model 2 (AUC = 0.853, 95% CI: 0.801–0.905; Figure 2E,F). Model 1 achieved higher overall accuracy (0.85 vs. 0.80) and balanced diagnostic metrics, with a sensitivity and specificity of 0.84, while model 2 had a sensitivity of 0.79 and a specificity of 0.81. The predictive reliability was further evidenced by positive predictive values (PPVs) of 0.84 (model 1) and 0.79 (model 2), alongside negative predictive values (NPVs) of 0.86 and 0.81. Calibration curves revealed significantly better agreement between predicted probabilities and observed outcomes for model 1 versus model 2, indicating enhanced probabilistic accuracy in risk stratification (Figure 2G,H). DCA demonstrated clinically meaningful net benefit for both models across threshold probabilities up to 80% (Figure 2I,J). This suggested that both models could be effectively integrated into clinical decision-making processes, offering substantial benefits in predicting PDAC risk.

### 3.3. Microbial Signature and CA19-9 Levels Improve Diagnostic Accuracy for PDAC

**Figure 3:**
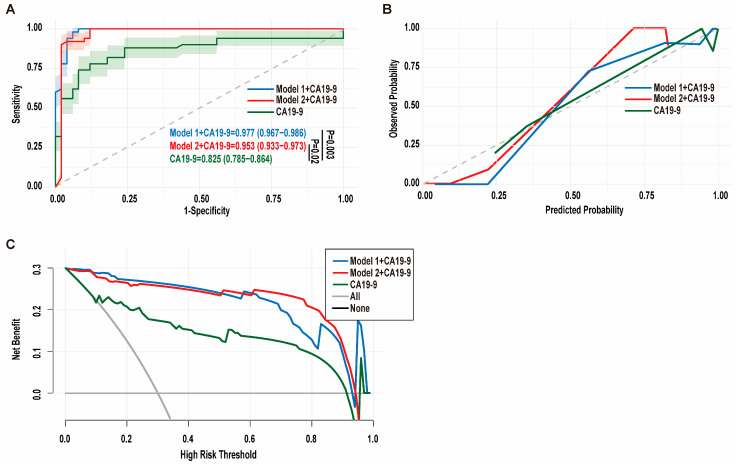
Microbial signatures and CA19-9 levels improve diagnostic accuracy for PDAC. (**A**) The ROC curves for CA19-9 alone and CA19-9 combined with microbial signatures from model 1 and model 2. The *p* values were obtained from Delong analysis. (**B**) The calibration curves for CA19-9 alone and CA19-9 combined with microbial signatures from model 1 and model 2. (**C**) DCA plots for CA19-9 alone and CA19-9 combined with microbial signatures from model 1 and model 2. ROC, receiver operating characteristic; AUC, area under the curve; DCA, decision curve analysis.

In routine clinical practice, CA19-9 stands as the sole blood-based biomarker for managing patients with PDAC. However, its limited sensitivity and specificity pose challenges to accurate detection in the general population. To overcome this limitation, we explored the diagnostic potential of combining microbial signatures with CA19-9. Serum CA19-9 levels were measured across all clinical samples, and their diagnostic performance was evaluated both independently and in combination with microbial signatures from model 1 or model 2. Notably, while CA19-9 alone achieved an AUC of 0.825 (95% CI: 0.785–0.864) across all PDAC stages, its integration with optimized signatures in model 1 or model 2 led to a significant enhancement in diagnostic accuracy. This improvement was evident in the increased AUC values of 0.977 (95% CI: 0.920–0.992, *p* = 0.003) for model 1 and 0.953 (95% CI: 0.905–0.978, *p* = 0.02) for model 2 (Figure 3A). In the entire cohort, CA19-9 alone had a sensitivity of 0.60 and a specificity of 0.94 for PDAC diagnosis. In contrast, model 1 achieved both a sensitivity and specificity of 0.94, and model 2 had a sensitivity and specificity of 0.92. These results clearly indicate that the combination of microbial signatures with CA19-9 significantly improves the screening capability for PDAC compared to CA19-9 alone.

Regarding diagnostic accuracy, our analysis showed that CA19-9 alone had an accuracy of 0.77 for distinguishing pancreatic cancer patients from healthy controls. However, when CA19-9 was combined with microbial signatures from model 1 and model 2, the accuracy improved significantly to 0.95 and 0.94, respectively. This enhancement highlights the added value of incorporating microbial features into the diagnostic framework. Moreover, calibration curves demonstrated that the combination of distinct microbial signatures preserved the favorable calibration and reliability of CA19-9 (Figure 3B). DCA revealed that, across a broad prevalence range of 0% to 90%, the combination of microbial signatures with CA19-9 achieved a substantial improvement in clinical net benefit compared to CA19-9 alone (Figure 3C). These data suggest that the combined approach has superior generalizability and clinical value. Collectively, these encouraging results highlight the potential of the microbial signature-based diagnostic strategy to complement CA19-9, thereby enhancing the diagnostic potential for PDAC screening.

### 3.4. Efficacy of the Microbial Signatures Across Different Confounders

To evaluate the robustness, clinical applicability, and translational potential of microbial biomarkers in routine diagnostics, we investigated the diagnostic efficacy of integrating microbial signatures with the conventional biomarker CA19-9 for PDAC detection across demographic subgroups stratified by age, gender, and BMI.

**Figure 4:**
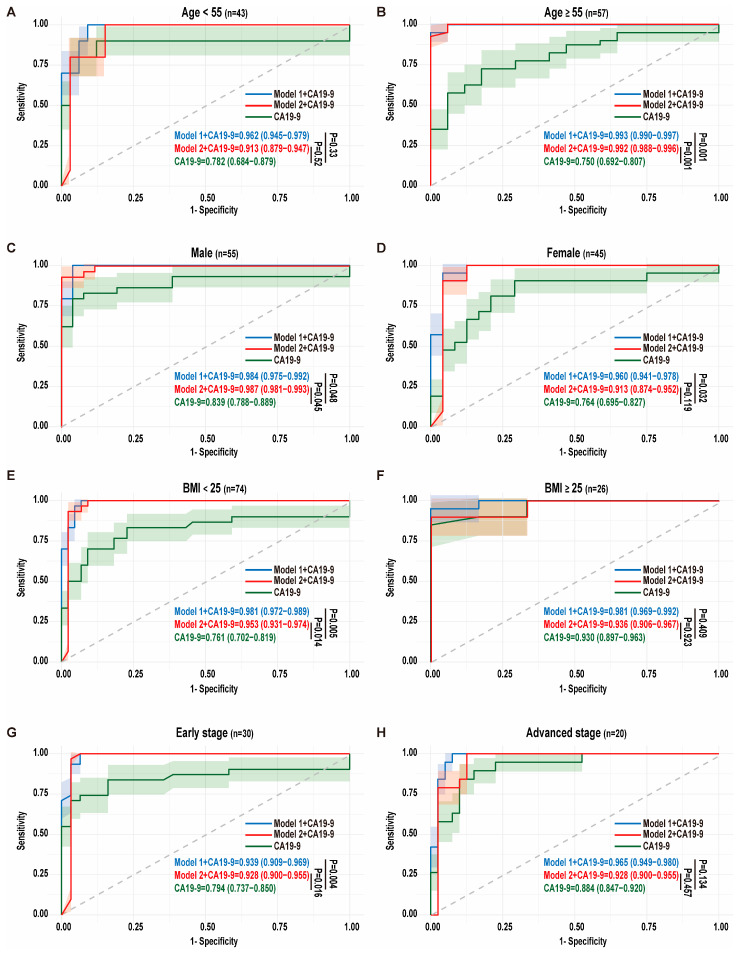
Efficacy of the microbial signatures across different confounders. (**A**,**B**) The ROC curves display the diagnostic performance of CA19-9 alone and CA19-9 combined with microbial features from model 1 and model 2 in the young group (n = 43) (**A**) and elderly people (n = 57) (**B**). (**C**,**D**) The ROC curves display the diagnostic performance of CA19-9 alone and CA19-9 combined with microbial features from model 1 and model 2 in males (n = 55) (**C**) and females (n = 45) (**D**). (**E**,**F**) The ROC curves display the diagnostic performance of CA19-9 alone and CA19-9 combined with microbial features from model 1 and model 2 in the normal-weight (n = 74) (**E**) and obese populations (n = 26) (**F**). (**G**,**H**) The ROC curves display the diagnostic performance of CA19-9 alone and CA19-9 combined with microbial features from model 1 and model 2 in the early-stage (n = 30) (**G**) and advanced-stage (n = 20) (**H**) patients with PDAC.

In younger individuals (<55 years), the integration of microbial signatures with CA19-9 yielded modest enhancements in diagnostic performance (model 1 + CA19-9: AUC = 0.962; model 2 + CA19-9: AUC = 0.913) compared to CA19-9 alone (AUC = 0.782), although these improvements did not reach statistical significance (*p* = 0.33 and 0.52). However, in the high-risk population, age ≥ 55 years, the combination of microbial models with CA19-9 demonstrated marked superiority. This synergistic approach achieved exceptional discriminative power, with AUC values of 0.993 (model 1 + CA19-9) and 0.992 (model 2 + CA19-9), significantly outperforming CA19-9 monotherapy (AUC = 0.750; *p* = 0.001) (Figure 4A,B). Furthermore, the integrated models substantially improved overall diagnostic accuracy (model 1 + CA19-9: 95%; model 2 + CA19-9: 95%) compared to standalone CA19-9 analysis (70%), underscoring their clinical utility in this critical demographic. These findings highlight the age-dependent diagnostic advantage of combining microbial biomarkers with conventional serological markers, particularly in older populations where PDAC risk is greatest.

Epidemiological evidence underscores a male predominance in PDAC incidence [30]. In males, the combined models demonstrated marked diagnostic superiority, achieving AUCs of 0.985 (model 1 + CA19-9) and 0.987 (model 2 + CA19-9), significantly outperforming CA19-9 monotherapy (*p* = 0.048 and 0.045, respectively). Diagnostic accuracy increased from 0.82 with CA19-9 alone to 0.95 with microbial integration. Similarly, in females, microbial supplementation enhanced diagnostic precision, particularly through genus-level features: model 1 synergized with CA19-9 to elevate AUC from 0.782 to 0.981 (*p* = 0.032) (Figure 4C,D).

Given that increased BMI is associated with a higher risk of PDAC [31], we conducted BMI-stratified evaluations. In normal-BMI individuals (<25 kg/m^2^), microbial integration with model 1 and model 2 achieved exceptional discriminative capacity (AUC = 0.981 and 0.953, respectively; accuracy = 95%), significantly surpassing CA19-9 alone (AUC = 0.761, *p* = 0.005 and 0.014; accuracy = 78%). However, in obese individuals (BMI ≥ 25 kg/m^2^), microbial augmentation showed numerically higher but statistically non-significant AUC improvements (model 1 + CA19-9: 0.981; model 2 + CA19-9: 0.936 vs. CA19-9: 0.930, *p* = 0.409 and 0.923), likely due to the already high diagnostic performance of CA19-9 in this population combined with the limited sample size of obese participants (n = 26), which may have reduced statistical power to detect modest effects (Figure 4E,F).

Considering the reported low positive predictive value of CA19-9 in early-stage asymptomatic PDAC [32], we further evaluated the discriminative ability of microbial features across tumor stages. In early-stage PDAC (TNM stages I and II), integrating CA19-9 with the nine microbial genera from model 1 significantly improved diagnostic performance, achieving an AUC of 0.939 (*p* = 0.004) compared to CA19-9 alone (AUC = 0.794; accuracy = 0.77). Similarly, combining CA19-9 with the eight microbial species from model 2 yielded an AUC of 0.928 (*p* = 0.016), with accuracies of 0.95 and 0.94 for the respective models. These results demonstrate that microbial signatures substantially enhance the diagnostic accuracy of CA19-9 in early-stage PDAC. In advanced-stage PDAC (TNM stages III and IV), the combination of CA19-9 with model 1 and model 2 also showed numerically improved AUCs compared to CA19-9 alone (AUC = 0.884; accuracy = 0.83), with AUCs of 0.965 and 0.928 (*p* = 0.134 and 0.457) and accuracies of 0.92 and 0.86, respectively (Figure 4G,H). The improvements in advanced stages did not reach statistical significance, potentially due to dominant CA19-9 elevation in late-stage disease, but the trend toward enhanced performance highlights the complementary role of microbial features across disease progression.

Collectively, these findings emphasize that while microbial-based predictive models exhibit robust standalone diagnostic potential, their integration with CA19-9 significantly elevates overall accuracy. This synergistic effect is particularly pronounced in early-stage PDAC, underscoring the clinical relevance of microbial signatures for improving early detection (Table 2).

## 4. Discussion

In this study, we characterized the gut microbiome of PDAC using shotgun metagenomic sequencing, revealing significant dysbiosis in the gut microbial composition compared to healthy controls. Using a random forest algorithm, we constructed microbial-feature-based diagnostic models for PDAC and demonstrated their strong diagnostic efficacy and accuracy through rigorous validation.

Metagenomic sequencing revealed distinct differences in the fecal microbiota between healthy individuals and patients with PDAC. Notably, commensal taxa with putative health-promoting roles, including *Blautia* (e.g., *Blautia wexlerae*, *Blautia obeum*) and *Bifidobacterium* (e.g., *Bifidobacterium longum*), were significantly depleted in PDACs. *Blautia* has been found to be reduced in abundance in severe acute pancreatitis [33], and *Bifidobacterium* has been shown to prevent acute pancreatitis by modulating pancreatic and systemic inflammation [34]. Conversely, the PDAC group exhibited an enrichment of *Bacteroides* and *Phocaeicola,* including species such as *Bacteroides_thetaiotaomicron, Bacteroides_uniformis, Bacteroides_stercoris, Bacteroides_fragilis, Phocaeicola_vulgatus,* and *Phocaeicola_coprocola.* Intratumoral *Bacteroides* have been associated with short-term survival in patients with PDAC [27], but research on their precise mechanistic roles in pancreatic carcinogenesis remains underexplored.

Beyond compositional shifts, our metagenomic analysis uncovered distinct functional disparities in microbial metabolic pathways between healthy controls and PDACs. Compared to the PDAC group, the gut microbiota of healthy individuals was enriched in pathways linked to amino acid metabolism, nucleotide biosynthesis, glycolysis, and purine metabolism. Specific amino acids, such as isoleucine and histidine, can induce selective toxicity to PDAC tumor cells [35]. Abnormal nucleotide metabolism fosters immunosuppression and therapy resistance [36]. Glycolysis and oxidative phosphorylation metabolism also play key roles in tumorigenesis [37]. We found that PDAC-associated microbiota exhibited pronounced enrichment of nucleotide sugar metabolism pathways. Aberrant nucleotide sugar metabolism may drive cancer progression by altering glycosylation patterns, thereby disrupting cell–cell communication, adhesion, and migration processes [38,39]. These findings highlight the potential of the gut microbiota and its metabolic pathways as diagnostic and therapeutic targets for PDAC. Further studies are needed to elucidate the specific mechanisms by which these microbial metabolic pathways influence PDAC development and progression.

Given the limited sensitivity and specificity of CA19-9 in detecting PDAC, particularly at early stages, the development of a microbiome-based diagnostic model holds substantial promise. This approach could offer a simple, noninvasive method for enhancing early detection rates, thereby improving patient outcomes.

The metagenomic classifiers developed at the genus and species levels (model 1 and model 2) demonstrated high discriminatory power, with AUC values of 0.923 and 0.853, respectively, alongside robust accuracy in the validation set. The enhanced diagnostic performance of genus-level features compared to species-level likely stems from greater taxonomic robustness against sequencing and database biases, functional conservation within bacterial genera that captures broader microbiome functional profiles, and improved technical reproducibility across sample processing methods. We developed both genus- and species-level models because while genus-level classification offers more stable diagnostic signatures, species-level resolution provides valuable mechanistic insights into specific microbial contributors to PDAC pathogenesis, which is particularly important given that certain pathogenic functions are often species-specific. The dual-model approach allows us to leverage genus-level stability for clinical applications while preserving species-level biological interpretability for future research directions. This strategy is especially relevant for metagenomic sequencing data, which inherently contain information at multiple taxonomic levels that can serve complementary purposes in biomarker development. Notably, the microbial taxa driving these models, though largely unexplored in PDAC, have been implicated in the pathogenesis of other malignancies. For instance, *Blautia*, which is depleted in PDAC but enriched in healthy controls, exerts tumor-suppressive effects in breast, bladder, and colorectal cancer, suggesting a potential conserved role in oncogenesis [23,40,41]. Conversely, *Clostridioides* and *Fusicatenibacter*, enriched in PDACs, are linked to colorectal cancer progression and lung adenocarcinoma metastasis, respectively [42,43]. *Coprobacillus* and *Pseudoflavonifractor* contribute to diagnostic classifiers or dysbiotic states in colorectal cancer [44,45]. The enrichment of *Desulfovibrio* is a known driver of hepatocellular carcinoma and colorectal cancer [46,47]. Of particular interest is the inclusion of *Siphoviridae*, a bacteriophage family that is enriched in PDAC-associated microbial signatures and has been found to be enriched in colorectal cancer [48,49]. While its role in pancreatic carcinogenesis remains uncharacterized, its presence raises intriguing questions about phage–bacterial interactions in shaping tumor-associated microbiomes. Further research is needed to elucidate the specific roles of these microbial features in PDAC and their potential as diagnostic or therapeutic targets.

Consistent with prior reports, CA19-9 alone exhibited suboptimal sensitivity (0.60) and accuracy (0.77) for PDAC diagnosis (AUC = 0.825). Strikingly, integrating microbial signatures from model 1 or model 2 with CA19-9 significantly enhanced diagnostic performance, achieving AUC values of 0.977 (*p* < 0.01) and 0.953 (*p* < 0.05), respectively, with accuracies of 0.94 and 0.92. This synergistic improvement underscores the added value of microbial features in compensating for the limitation of CA19-9, particularly in early-stage detection. The superior clinical net benefit of the combined models across a wide prevalence range further supports their translational potential as noninvasive screening tools.

Despite the multifactorial nature of PDAC diagnosis, the predictive performance of our combined microbial-CA19-9 models remained consistently high across subgroups stratified by age, sex, and BMI, underscoring their generalizability and stability in diverse populations. We observed that microbial biomarkers exhibit enhanced diagnostic performance in older populations. This superior performance likely stems from age-related biological changes including cumulative microbial dysbiosis from prolonged exposure to risk factors, immunosenescence that alters host–microbe interactions, and metabolic shifts that amplify microbial contributions to tumorigenesis. These aging-associated processes enhance the detectability of cancer-related microbial signatures in older individuals where conventional markers often show limited sensitivity. In males, the combined models demonstrated marked diagnostic superiority, likely reflecting gender-specific microbiota variations. European multicenter studies reveal male PDAC patients exhibit higher *Bacteroides* and *Prevotella* abundance than females [50]. Smoking, a male-predominant PDAC risk factor, further shapes tumor-associated microbiomes to enhance carcinogenic potential [51,52]. These synergistic effects may amplify the diagnostic fidelity of microbial signatures in male patients. We found that in individuals with obesity (BMI ≥ 25), microbial features failed to significantly enhance the diagnostic efficacy of CA19-9. Obesity may affect the diversity and stability of the gut microbiota. For example, obese adults have significantly lower α-diversity (Shannon index) compared to non-obese adults. The abundance of *Firmicutes* is higher in obese adults than in non-obese adults, while the abundance of *Bacteroidetes* is significantly reduced [53]. Additionally, the dietary habits of obese individuals may lead to changes in the composition and function of the gut microbiota. Diets rich in carbohydrates or high in fat can enhance the ability of *Firmicutes* to extract energy from the diet, which in turn can affect the efficacy of microbial biomarkers. Obese individuals often suffer from metabolic syndrome, including insulin resistance, hyperglycemia, and dyslipidemia. These metabolic changes may alter the metabolic activities of the gut microbiota, thereby affecting the diagnostic performance of microbial biomarkers [19,54,55]. Moreover, the limited sample size of this population may have affected the statistical accuracy; larger studies with stratification by BMI are needed to clarify these effects. Notably, CA19-9 alone demonstrated limited diagnostic efficacy for early-stage PDAC (AUC = 0.794, accuracy = 0.77), aligning with its well-documented shortcomings in detecting early disease. However, integrating microbial signatures from model 1 and model 2 with CA19-9 markedly improved diagnostic performance for early-stage PDAC, achieving AUC values of 0.939 and 0.928 and accuracies of 0.95 and 0.94, respectively. The enhanced diagnostic performance of the combined model, integrating microbial signatures with CA19-9, underscores the potential of microbial markers as valuable complements for early PDAC detection. Early detection is pivotal for improving outcomes, as early-stage PDAC is more amenable to resection and treatment. This integrated approach can facilitate personalized diagnostic strategies, identifying patients who may benefit from intensified surveillance or early intervention. The addition of microbial signatures can mitigate the limitations of CA19-9 in sensitivity and specificity, reducing false-negative results and enhancing overall diagnostic efficacy. The gut microbial composition may change in early disease phases, modulating PDAC progression via the generation of bioactive metabolites. For instance, butyrate, an SCFA derived from bacterial metabolism of dietary fiber, has been shown to inhibit PDAC cell proliferation and promote differentiation [15]. Microbial signatures can also modulate the host immune response, stimulating antitumor immunity or dampening pro-inflammatory pathways, which is crucial for early detection and treatment [13]. These results collectively demonstrate the feasibility of developing a comprehensive, specific, and reproducible predictive model for early PDAC detection based on noninvasive gut microbiome analysis. However, in advanced-stage PDAC, microbial signatures failed to significantly improve diagnostic performance compared to CA19-9 alone. This may be due to the marked elevation of CA19-9 in late-stage disease, which can overshadow microbial signals, as observed in the European EPIC cohort study [56]. Additionally, extensive fibrotic remodeling in advanced PDAC may reduce microbial tumor colonization, diminishing detectable signatures. Despite the lack of statistical significance, combining microbial signatures with CA19-9 can still offer valuable insights for advanced-stage PDAC, potentially identifying patients who may benefit from more aggressive treatment strategies. Future research should explore integrating microbial signatures with other emerging biomarkers, such as circulating tumor DNA or inflammatory markers, to further enhance diagnostic accuracy.

This study has several limitations. Firstly, as this was a single-center investigation with a moderate sample size (50 patients with PDAC and 50 controls), our findings may have limited generalizability and statistical power to detect subtler microbial differences. While our matched cohort design controls for key confounders, we are actively addressing these limitations by establishing an international multicenter validation cohort encompassing diverse geographic regions (Asia, Europe, North America), ethnicities (Asian, Caucasian, African American), and dietary patterns (Western, Mediterranean, high-fiber, high-fat, and ketogenic diets) to evaluate model performance across genetically distinct populations. These planned validations will systematically assess whether the microbial signatures require population-specific calibration or demonstrate universal applicability, particularly for early-stage detection where microbiome-based diagnostics could offer the greatest clinical impact. External validation through these larger, biologically heterogeneous cohorts will be essential to confirm these microbial signatures and enhance the reliability of our predictive models for global clinical application. Secondly, while metagenomic sequencing is costly, it provides high-resolution insights into the gastrointestinal microbiota, enabling species- and strain-level identification and functional analysis. Moving forward, we aim to explore whether these microbial features can serve as prognostic biomarkers for chemotherapy or immunotherapy on PDAC. Additionally, future studies should integrate metabolomic data to better understand the role of microbiota in PDAC development and progression, potentially uncovering new diagnostic and therapeutic targets.

In conclusion, our study shows that microbial features have strong diagnostic efficacy, accuracy, and robustness for PDAC. Combining microbial biomarkers with traditional markers like CA19-9 enhances early detection. This noninvasive approach could become a powerful screening tool in clinical practice and advance our understanding of the microbiome’s role in PDAC. Future work should focus on validating these findings in larger cohorts and exploring the mechanisms underlying the microbiome’s involvement in PDAC development.

## Figures and Tables

**Table 1:** Demographic and clinical characteristics of patients with PDAC and controls.

Characteristics	PDAC	Controls	*p*-Value
Total samples (feces, n)	50	50	NA
Sex (male/female, n)	29/21	26/24	0.546
Age, years, (mean ± SD)	63.46 ± 11.03	62.0 ± 8.89	0.468
BMI, kg/m^2^, (mean ± SD)	23.68 ± 3.38	22.54 ± 2.28	0.090
Smoking, n (%)	11 (22%)	8 (16%)	0.444
Alcohol, n (%)	4 (8%)	4 (8%)	1.000
Diabetes mellitus, n (%)	5 (10%)	7 (14%)	0.538
CA19-9, U/mL, median (IQR)	66.4 (17.35–171.8)	8.3 (5.3–11.18)	<0.0001
UICC classification			
IA, n (%)	8 (16%)	NA	NA
IB, n (%)	11 (22%)	NA	NA
IIA, n (%)	3 (6%)	NA	NA
IIB, n (%)	8 (16%)	NA	NA
III, n (%)	12 (24%)	NA	NA
IV, n (%)	8 (16%)	NA	NA
Tumor stage			
T1, n (%)	11 (22%)	NA	NA
T2, n (%)	20 (40%)	NA	NA
T3, n (%)	9 (18%)	NA	NA
T4, n (%)	10 (20%)	NA	NA
Lymph node invasion			
N0, n (%)	28 (56%)	NA	NA
N1, n (%)	9 (18%)	NA	NA
N2, n (%)	13 (26%)	NA	NA
Metastases			
M0, n (%)	42 (84%)	NA	NA
M1, n (%)	8 (16%)	NA	NA

PDAC, pancreatic ductal adenocarcinoma; IQR, interquartile range; SD, standard deviation; BMI, body mass index; NA, Not Applicable.

**Table 2:** Performance characteristics of microbial signatures and CA19-9 alone.

	Model	AUC	Accuracy	Sensitivity	Specificity	PPV	NPV
Age < 55 (n = 43)	Model 1 + CA19-9	0.96	0.93	0.90	0.94	0.82	0.97
	Model 2 + CA19-9	0.91	0.88	0.80	0.91	0.73	0.94
	CA19-9 alone	0.78	0.88	0.60	0.97	0.86	0.89
Age ≥ 55 (n = 57)	Model 1 + CA19-9	0.99	0.95	0.95	0.95	0.97	0.89
	Model 2 + CA19-9	0.99	0.95	0.95	0.94	0.97	0.89
	CA19-9 alone	0.75	0.70	0.60	0.88	0.92	0.48
Male (n = 55)	Model 1 + CA19-9	0.98	0.95	0.93	0.96	0.96	0.93
	Model 2 + CA19-9	0.98	0.95	0.93	0.96	0.96	0.93
	CA19-9 alone	0.84	0.82	0.69	0.96	0.95	0.74
Female (n = 45)	Model 1 + CA19-9	0.96	0.93	0.95	0.92	0.91	0.96
	Model 2 + CA19-9	0.91	0.89	0.91	0.88	0.86	0.91
	CA19-9 alone	0.76	0.72	0.64	0.92	0.83	0.67
BMI < 25 (n = 74)	Model 1 + CA19-9	0.98	0.95	0.95	0.93	0.93	0.95
	Model 2 + CA19-9	0.95	0.95	0.93	0.95	0.93	0.95
	CA19-9 alone	0.76	0.78	0.67	0.92	0.85	0.76
BMI ≥ 25 (n = 26)	Model 1 + CA19-9	0.98	0.92	0.95	0.83	0.95	0.83
	Model 2 + CA19-9	0.94	0.85	0.90	0.67	0.90	0.67
	CA19-9 alone	0.93	0.73	0.65	0.98	0.98	0.46
Early stage (n = 30)	Model 1 + CA19-9	0.94	0.95	0.97	0.90	0.91	0.98
	Model 2 + CA19-9	0.93	0.94	0.98	0.87	0.89	0.98
	CA19-9 alone	0.79	0.77	0.60	0.97	0.95	0.70
Advanced stage (n = 20)	Model 1 + CA19-9	0.97	0.92	0.84	0.95	0.89	0.93
	Model 2 + CA19-9	0.93	0.86	0.79	0.90	0.79	0.90
	CA19-9 alone	0.88	0.83	0.58	0.8	0.80	0.84

CA19-9: Carbohydrate Antigen 199; AUC: area under the curve; PPV: positive predictive value; NPV: negative predictive value.

## Data Availability

The accession number for the sequencing data reported in this paper is SRA: PRJNA 1196505. Other data that support the findings of this study are available from the corresponding author upon reasonable request.
